# Management of orofacial clefts in Africa, insufficient management centers, and workforce

**DOI:** 10.1097/JS9.0000000000000139

**Published:** 2023-03-03

**Authors:** Andrew A. Wireko, Favour T. Adebusoye, Pearl O. Tenkorang, Aashna Mehta, Mubarak J. Mustapha, Anastasia F. Debrah, Rohan Yarlagadda, Owusu Y. Asieduwaa, Toufik Abdul-Rahman, Oti N. Victor, Vladyslav Sikora, Marios Papadakis

**Affiliations:** a Sumy State University, Sumy; b Kyiv Medical University, Polish Campus, Bytom, Poland; c University of Ghana Medical School, Accra; d Faculty of Pharmacy and Pharmaceutical Sciences, Kwame Nkrumah University of Science and Technology, Kumasi, Ghana; e Faculty of Medicine, University of Debrecen, Debrecen, Hungary; f Faculty of Basic Medical Sciences, University of Ilorin, Ilorin, Nigeria; g Rowan University School of Osteopathic Medicine, Stratford, New Jersey, USA; h Department of Surgery II, University Hospital Witten-Herdecke, University of Witten-Herdecke, Wuppertal, Germany

An orofacial cleft (OFC) is a gap in the lip, upper jaw, and/or palate. OFCs are relatively common congenital deformities with both genetic and environmental risk factors^1^. While the primary causes of OFCs are still under investigation, genetics may play a significant role. Furthermore, nongenetic risk factors such as folic acid deficiency, maternal smoking, maternal alcohol consumption, and maternal drug use have been linked to OFCs in neonates^2^. OFC patients may develop several complications, including recurring ear infections, dental issues, and hearing loss. It is widely assumed that the global prevalence of OFCs is one in 600–800 live births^2^. Data on the epidemiology of OFCs in various populations varied depending on geographical location or ethnicity^3^. In low- and middle-income countries (LMICs), 1 in 730 children is born with a cleft lip or palate^3^. The availability of multidisciplinary OFC teams has resulted in a decrease in the incidence of OFCs in middle- and high-income countries, according to several studies. Although it is estimated that OFC incidence is low in LMICs, disability remains high in marginalized communities due to limited access to treatment^4^.

In Africa, the estimated birth prevalence of OFCs is 0.5 per 1000 live births or 1 : 2000 infants^5^. The prevalence of OFCs in various African communities varies greatly, ranging from 0.3/1000 in Nigeria to 1.65/1000 in Kenya. It has been reported in several papers that the prevalence of OFCs is low in developing nations^6^,^7^ but this may be inaccurate due to the lack of precise and comprehensive studies to thoroughly assess the case in LMICs. Many patients with OFCs are rarely reported in hospitals, particularly in Africa, for a variety of reasons such as societal neglect and fear of surgery-related mortality. The precise records of antenatal care, births, and deaths are not available, making findings from most studies on OFCs in Africa incomplete^8^. Purchases of over-the-counter drugs, such as opioids are common in Africa, and a few studies have indicated that they may be a risk factor for congenital anomalies^8^. Another challenge Africa faces in terms of OFC risks is stringent customs and norms that encourage the use of naturopathic medicine, which is primarily made up of herbs from unknown sources and contains teratogenic substances, increasing the likelihood of OFC formation in offspring^7^,^8^.

This editorial discusses the ailing state of OFC management in Africa by reflecting on progress made in the past decades and putting forward possible recommendations.

## Progress over the decades

Over the last two decades, there has been tremendous progress in the management of OFCs in low-income nations, particularly in Africa. International organizations such as the Smile Train Organization and Smile South Africa have intervened to treat people with OFCs, with the assistance of some countries’ governments^7^,^9^. In the roughly 15 years since the Smile Train Organization began operations around the world, they have been able to treat over 1 000 000 people, with the majority of their operations taking place in Africa^7^. Recent studies show that the incidence of OFCs has decreased in recent years, demonstrating the substantial impact of these international community efforts and the government’s partnerships. These were accomplished by constructing more management centers, training more healthcare workers, implementing a multidisciplinary team approach, providing nutritional counseling, free surgical care, serious awareness campaigns, and a variety of other initiatives^10^,^11^.

## OFC management gaps in Africa

For decades, the African perception of OFCs has been a serious challenge, particularly for OFC patients and their families, as well as for OFC management. The diverse contributions of societal attitude, culture, and tradition to cleft palate lead to additional psychological challenges, social disengagement, and aggression in OFC patients and families^12^. Due to the severe societal stigmas, both children and adults with OFCs may suffer from severe psychological trauma.

Adult presentation of OFCs is a major challenge in most African nations. Under normal circumstances, rehabilitation is more successful when OFC surgical treatments are performed in the very early years of children that are below 2 years^9^. Due to high illiteracy rates, the general public is unaware that OFC is treatable. Many parents have avoided surgical procedures and have refused treatment for cultural and superstitious reasons^7^,^11^. In addition, poverty is a major contributor to Africa’s poor management of OFCs. Forty percent of Nigeria’s 206 million people live in abject poverty, with 83 million impoverished Nigerians unable to afford health insurance^13^. As a result, many OFC patients are not identified for treatment due to the high cost of surgery. Furthermore, given that the private sector provides most of Africa’s health services, the high cost of healthcare services frequently prevents most patients from seeking treatment^13^. Also, prior unpleasant hospital or surgery-related experiences may deter patients from future healthcare engagement.

Africa has the fewest specialist surgeons and anesthetists, with only 0.5 surgeons and 0.1 anesthetists serving every 100 000 people^14^. Plastic surgery supply in Low Income Country (LICs) is estimated to be 0.0073 plastic surgeons per 100 000 population, compared to 2.18 in the USA^15^. Children with OFC frequently struggle with adequate nutrition, speaking, tooth development, ear infections, and other issues, making them more vulnerable than other children and contributing to their high mortality rate in these low-income nations^16^. Despite this high mortality burden that OFC poses in LICs, reconstructive surgeons are underrepresented, indicating a predominance of difficulties related to facial clefts treatment^15^. Patients across the continent lack the opportunity to receive standard of care treatment due to a scarcity of surgical services and challenges in accessing the few paid local surgeons in cities.

Nonsurgical challenges, such as attending nonspecialists’ responsibilities, limited funds to address patients’ nonsurgical treatment demands, and a lack of commitment on the part of some nonsurgical team members, all pose challenges to efficient OFCs care^11^. Patients’ misconceptions about the treatment, a proclivity for delayed diagnosis, poverty, failure to comply with postoperative follow-up appointments, and inability to complete rehabilitation programs are all exacerbating issues^7^,^11^. Other patient-related challenges include a low tolerance for satisfaction with functional and esthetic outcomes, as well as some distressing intraoperative events that developed and caused some cases to be postponed or canceled^11^. Case cancellation can happen for a variety of reasons, such as respiratory illness, cardiac abnormality, a lack of theater space, delayed recuperation, industrial disruption, and so on. Case scheduling issues caused by competing interests of other patients, as well as a lack of beds, are examples of hospital-related challenges^7^,^11^. The majority of the continent’s healthcare system is entirely self-funded, and the few surgical services that are available are disproportionately concentrated in urban areas, necessitating costly travel and frequently resulting in huge bills that establish a seemingly insurmountable financial barrier.

## Recommendations

The prevalence of untreated OFCs in Africa is alarming, and effective measures are needed to prevent new cases and manage existing ones. Inadequate health infrastructure in the form of management facilities and health professionals is one of the factors contributing to the high rate of OFCs in Africa. Thus, African governments should build more accessible clinic facilities to ensure the availability of management facilities. African nations should train more diverse therapy teams, including nurses, plastic surgeons, otorhinolaryngologists, pediatric cardiologists, speech therapists, dietitians, pediatric dental surgeons, and maxillofacial surgery specialists. Most importantly, these new healthcare facilities and personnel should be distributed equitably across all regions, especially rural areas, where the majority of these unaddressed complex cases may be found. African governments should strive to maintain good living and working conditions for the health professionals in charge of these OFC cases in order to prevent the brain drain of highly skilled African healthcare workers. Newborns must be examined by specialists to ensure that no deforming conditions, such as OFCs, are present and that appropriate surgical interventions are applied if one were to be identified. Healthcare professionals should be encouraged to look for signs of such conditions as early as possible to prevent future complications.

Developing well-structured innovative prevention strategies is one of the most important ways to reduce birth deformities like OFCs. There should be equitable vaccination strategies for the African population, particularly pregnant women considering that many congenital cases, such as OFC, are caused by viral infections. Furthermore, African governments and other international organizations should prioritize public education about the importance of folic acid supplementation during pregnancy in preventing OFCs among other congenital complications. In high-risk households, family planning in the form of genetic counseling and preconception screening should be carefully prioritized. There should be low-cost and easily accessible antenatal and postnatal services to ensure proper monitoring of unborn and born infants and effective therapeutic measures to manage OFCs when necessary. Improved nutrition knowledge and education on OFC-induced variables such as naturopathic medicine, secondhand smoking, corticosteroids, radiation exposure, viral infections, and diabetes during pregnancy should be incorporated into prenatal programs.

In the next few years, it is clear that many more reconstructive surgeons will need to be involved in the push for the growth in available healthcare practitioners. This will need a coordinated effort on the part of the government, plastic surgery community, and health systems to provide such training opportunities, as well as for international nongovernmental organizations to incorporate into current LIC training programs with the goal of generating LIC plastic surgeons. The inclusion of surgeons in these processes combined with policies to support the OFCs surgery workforce may help to improve access and quality of reconstructive services^15^. To improve the accessibility of treatment of OFCs, plastic surgery objectives should be integrated into National Health Plans. Recognizing and strengthening the local workforce entails estimating the number of surgeons working and living in LMICs and offering assistance with training, facilities, and financial compensation as needed^15^.

National Surgical Anesthesia and Obstetric Plans have evolved as a method of systematically addressing gaps in access to surgical care in LMICs through informed national policy and relying on scale-up of the local surgical workforce to improve global access to quality surgical care. African countries that effectively implement these NSAOPs will undoubtedly improve their surgical delivery.

Finally, more research should be conducted to develop better methods of managing OFCs in Africa, as there is very little data to critically assess surgical cases on the continent. Future research should focus on determining the extent to which nonplastic surgeons share tasks and evaluating the effectiveness of alternative plastic surgery treatment models in low-resource countries.

## Ethical approval

Not applicable.

## Consent for publication

Not applicable.

## Sources of funding

None.

## Author contributions

W.A.A.: conceptualization ideas. All authors involved in data curation, writing of initial draft, review and editing, and final approval of the manuscript.

## Conflicts of interest disclosure

Authors declare no conflict of interest.

## Research registration unique identifying number (UIN)

None.

## Guarantor

Wireko Andrew Awuah.

## Availability of supporting data

No new data generated.
